# Ethical considerations in decentralizing antivenom treatment in indigenous territories of the Brazilian Amazonia

**DOI:** 10.1371/journal.pntd.0014577

**Published:** 2026-07-27

**Authors:** Thiago Serrão-Pinto, Felipe Murta, Vinicius Machado, Altair Seabra de Farias, Elder Augusto Guimarães Figueira, Érica Cristina da Silva Chagas, Luana Souza Habibe, Flávio Santos Dourado, Lucia Montebello, Nicholas R. Casewell, David G. Lalloo, Fernando Almeida-Val, Marcus Lacerda, Joao Ricardo Nickenig Vissoci, Charles J. Gerardo, Fan Hui Wen, Jacqueline Sachett, Wuelton Monteiro

**Affiliations:** 1 School of Health Sciences, Universidade do Estado do Amazonas, Manaus, Brazil; 2 Department of Teaching and Research, Fundação de Medicina Tropical Dr. Heitor Vieira Dourado, Manaus, Brazil; 3 Department of Environmental Surveillance, Fundação de Vigilância em Saúde do Amazonas Dra. Rosemary Costa Pinto, Manaus, Brazil; 4 Indigenous Health Secretariat, Ministry of Health, Brasília, Brazil; 5 Secretariat for Health and Environmental Surveillance, Ministry of Health, Brasília, Brazil; 6 Centre for Snakebite Research and Interventions, Liverpool School of Tropical Medicine, Pembroke Place, Liverpool, United Kingdom; 7 Department of Tropical Disease Biology, Liverpool School of Tropical Medicine, Pembroke Place, Liverpool, United Kingdom; 8 Medicine School, Federal University of Amazonas, Manaus, Brazil; 9 Duke Global Health Institute, Duke University, Durham, North Carolina, United States of America; 10 Department of Emergency Medicine, Duke University, Durham, North Carolina, United States of America; 11 Bioindustrial Center, Butantan Institute, São Paulo, Brazil; Universidad de Costa Rica, COSTA RICA

## Abstract

Snakebite envenoming disproportionately affects Indigenous populations in the Brazilian Amazon, where centralized healthcare systems limit timely access to antivenom. The recently implemented SAVING Program has decentralized antivenom delivery to Indigenous primary healthcare facilities, improving access and clinical outcomes. This article examines the ethical considerations encountered and the approaches adopted to address these challenges. Using the WHO/TDR framework for ethics in implementation research as a starting point, we continuously adapted to ethical issues arising during the planning, implementation, and dissemination of the program. The analysis was informed by focus groups and stakeholder discussions involving Indigenous leaders, patients, healthcare professionals, health managers, policymakers, and representatives of Indigenous governance structures. Key themes included community and stakeholder responsiveness, informed consent, balancing risks and benefits, culturally appropriate standards of care, data governance, sustainability, and benefit sharing. Decentralizing antivenom delivery can improve equitable access to lifesaving treatment while strengthening Indigenous health systems. To date, the program has demonstrated the feasibility of building sustainable partnerships with Indigenous communities, enhancing acceptance and equity. The ethical lessons from the SAVING Program may provide practical guidance for implementing health innovations in Indigenous and other historically underserved communities.

## Introduction

In Brazil, snakebite envenoming disproportionately affects rural and Indigenous populations, where delays in accessing antivenom significantly increase morbidity and mortality [1]. Centralized models of healthcare delivery, in which health technologies are available exclusively in hospital settings located in urban areas, as is the case with antivenom treatment in Brazil, are incompatible with the geographic and socio-cultural realities of Indigenous territories [2]. Decentralization of antivenom delivery has emerged as a promising strategy to address these barriers [3]. Implementation in such settings has proven both effective and feasible [4]; however, important ethical aspects have emerged during the process that warrant careful consideration and should be brought to the attention of the research and policymaking communities. This reflection aims to inform ethically guided robust strategies for long-term sustainability and scaling up implementation within Indigenous and other minority populations.

**Fig 1:**
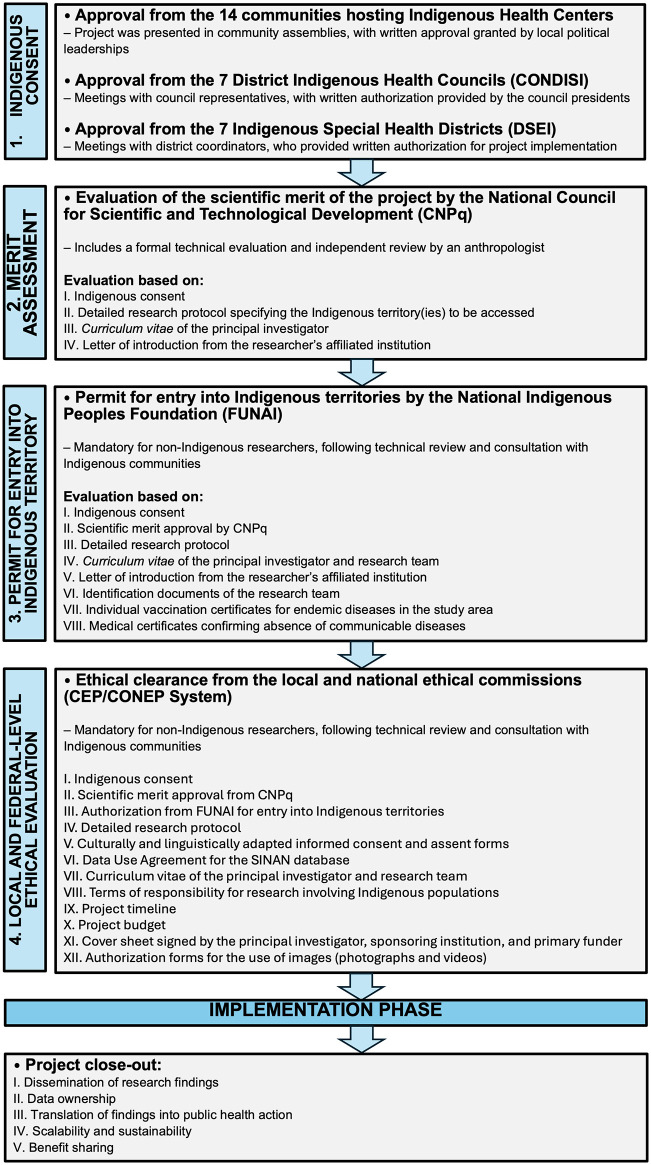
Ethical clearance pathway for research involving Indigenous populations in Brazil. Sequential approval process required for the SAVING Program, including Indigenous community consent, scientific merit review, authorization for entry into Indigenous territories, and ethical clearance at local and national levels. The figure outlines key institutions involved, and the documentation required at each stage prior to implementation.

In Brazil, research involving Indigenous populations requires a sequential approval process, including scientific merit review by the National Council for Scientific and Technological Development (CNPq) and authorization from the National Indigenous Peoples Foundation (FUNAI) for entry into Indigenous territories. The Brazilian National Research Ethics System (CONEP) also requires free, prior, and informed consent from Indigenous leadership, along with approvals from District Indigenous Health Councils and Indigenous Special Health Districts. Final authorization by the National Commission for Research Ethics is required before project initiation (Fig 1).

Here, we examine the ethical challenges associated with decentralizing antivenom treatment in Indigenous territories, drawing on empirical observations from Indigenous healers, patients, healthcare providers and managers. Data were collected from November 2024 to March 2025 through focus groups conducted by experienced researchers. A final discussion of the findings was held in March 2025 in Manaus with participants from the *Fundação de Vigilância em Saúde Dra. Rosemary Costa Pinto* (FVS-RCP, the institution responsible for snakebites surveillance and antivenom distribution in the Amazonas state), Venomous Animals Technical Group and Indigenous Health Secretariat of the Brazilian Ministry of Health, *Fundação de Medicina Tropical Dr. Heitor Vieira Dourado* (FMT-HVD, tertiary hospital serving as reference for snakebite treatment in the state), representatives of the seven Special Indigenous Health Districts (SIHD), and of the District Indigenous Health Council (CONDISI). Findings are presented according to the domains of the WHO/TDR ethical framework for implementation research [5].

### The SAVING Program

The SAVING Program was implemented in fourteen Indigenous Health Poles in the state of Amazonas, Brazil, using a quasi-experimental pre-post design to estimate effectiveness based on time to antivenom administration, clinical severity, case-fatality, along with early adverse reactions and implementation outcomes [4]. The intervention significantly increased timely antivenom administration, decreased severity of envenomings and reduced deaths, with few and manageable adverse reactions. High acceptability, protocol adherence, and trust in local services were reported. These findings support decentralized antivenom delivery as a safe and effective strategy to improve access and outcomes in remote Indigenous settings.

The SAVING Program underwent the complex ethical approval process over approximately one year, ensuring alignment with regulatory requirements. The Amazonas Bipartite Intermanagerial Commission—the collegiate body responsible for coordinating health system governance at state and municipal levels—formally approved the SAVING Program.

This Policy Platform uses the decentralization of antivenom treatment in Indigenous territories as a real-world case study to examine ethical considerations throughout the planning, implementation, and post-implementation phases of implementation research. We illustrate how internationally recognized ethical principles can be operationalized when implementing complex health interventions in low-resource and marginalized settings. For each phase, we discuss the ethical challenges that emerged, the strategies adopted to address them, and the lessons learned, concluding with practical recommendations for researchers conducting implementation research with Indigenous and other historically marginalized populations.

### Ethical statement

This protocol was approved by the National Research Ethics Commission (approval number 4,993,083/2020). Written informed consent was obtained from all participants.

## Planning phase

### Responsiveness to local needs and priorities

The SAVING Program was derived after a longstanding demand from Indigenous leaders, formally expressed in 2019 through a joint request for access to antivenom within community health centers [3]. Snakebite envenoming remains a major yet underrecognized burden among Indigenous populations in the Brazilian Amazon, where access to antivenom is limited to distant urban hospitals. This centralized model leads to delayed treatment, severe outcomes, and preventable deaths [1,6], compounded by underreporting and fragmented care pathways linked to geographic barriers and reliance on traditional medicine [3,7]. Limited use of hospital services reflects not rejection of biomedical care, but longstanding cultural and logistical constraints [8,9]. For decades, Indigenous communities and frontline health workers have demanded access to antivenom within their territories. The SAVING Program responds to this need by decentralizing services and strengthening local capacity to improve timely and culturally appropriate care, in alignment with the National Policy for the Health Care of Indigenous Peoples [10].

### Equipoise

Delays in accessing hospital-based treatment are associated with preventable morbidity and mortality, while administering antivenom in remote settings raises concerns about managing adverse reactions, trained personnel, and infrastructure. Although antivenom is an effective and established treatment [11], evidence is derived from hospital settings where adverse reactions can be managed more promptly in case of severe allergic reactions [12]. This study represents the first evaluations of antivenom use in primary care units within Indigenous territories, where conditions differ substantially. In this new care setting, the duty to ‘do no harm’ of any intervention is not clear to be achieved. Considering the high-quality antivenoms produced in Brazil, policy makers and health care professionals reported resorting to suboptimal alternatives due to lack of access, suggesting that maintaining the centralized model may cause greater harm [4], thus justifying this research.

### Study design

The study design prioritized ethical feasibility over strict experimental control. A randomized trial would require withholding local access to antivenom and maintaining referral to distant hospitals despite known delays and worse outcomes. We therefore used a quasi-experimental pre–post design, comparing outcomes before and after the SAVING Program using *Sistema de Informação de Agravos de Notificação* (SINAN; Notifiable Diseases Information System) database, where snakebite is a notifiable condition in Brazil [7]. This approach avoids denying beneficial care and enables evaluation under real-world conditions in Indigenous territories. Although more prone to confounding, it provides high external validity and is ethically appropriate where randomization is not feasible [13].

### Managers, health staff and community engagement

A co-development process brought together key institutions, including FVS-RCP, Venomous Animals Technical Group and Indigenous Health Secretariat of the Brazilian Ministry of Health, FMT-HVD, representatives of the seven SIHD, and experts of the Butantan Institute (the major antivenom producer in Brazil). Stakeholder engagement was embedded from the outset through close collaboration between institutions, researchers, and health system managers across all levels. This co-development process ensured alignment with national policies and operational realities. The CONDISI provided formal approval for the project and was actively involved throughout its development and implementation. As a deliberative and consultative body within the Brazilian Unified Health System, CONDISI ensures social control by including Indigenous representatives, health workers, and managers in decision-making processes. Its participation was critical to ensure that the project aligned with community priorities, upheld principles of equity, and maintained legitimacy within Indigenous health governance structures.

Frontline health care professionals from Indigenous Health Poles were engaged through structured discussions prior to implementation to identify operational challenges and training needs. They contributed to adapting clinical protocols and defining care workflows. This process also enabled co-development of the intervention, strengthening its feasibility and alignment with care setting conditions.

Community engagement was designed to ensure meaningful participation of Indigenous communities, including informing potential users of antivenoms, supporting shared decision-making, and strengthening community-led initiatives. Strategies within the SAVING Program included pre-scheduled town halls, focus groups, and interactive workshops conducted with community leaders and traditional healers (*pajés* or shamans). All activities were supported by bilingual interpreters and culturally adapted materials to ensure clear communication. This approach aimed to improve acceptability, adoption, and integration of the intervention within local care practices.

### Balance between risks and benefits

The decentralization of antivenom to Indigenous health units is expected to improve key indicators, including reduced time to treatment, decreased severity, and increased acceptability and user trust. However, it introduces operational risks related to adverse reactions management, cold chain integrity, and availability of trained personnel. These risks were weighed against the impact of delayed treatment under the centralized model, which is associated with worse outcomes and reduced system effectiveness. Mitigation measures included restricting implementation to units meeting minimum infrastructure and staffing requirements, supported by a standardized accreditation checklist [14] and targeted training based on a culturally adapted clinical guideline [15]. This framework supports a favorable operational balance, with gains in access and timeliness outweighing controlled risks.

## Implementation phase

### Autonomy and informed consent

Although the intervention increased users’ practical autonomy, important limits remain in patients’ therapeutic choices, particularly in groups where traditional healers (pajés) play a central role in deciding whether and when to seek biomedical care. Providers recognize that these practices may delay antivenom administration and worsen outcomes, yet there is broad consensus on respecting them unless clearly harmful. To navigate this, providers often adapt care by administering antivenom in villages, outside the health care facility despite protocol deviations, to ensure timely treatment. These dynamics reflect ethical tensions and trade-offs inherent to decentralization, notably between autonomy and cultural respect, and between protocol adherence and contextual adaptability. Care delivery is frequently negotiated with community leaders, healers, and families, with trust playing a key role in acceptance of treatment. Decision-making is thus collective, reinforcing that autonomy in this context is relational and shaped by local authority structures. Given the tension between autonomy and protocol adherence, further research should be developed to ensure safe protocols are embedded within cultural practices that maintains autonomy. A consideration that is critical to engage with this population, historically explored and denied its autonomy.

The absence of physicians during certain periods posed an ethical challenge for nurses, who lack the legal authority to prescribe antivenom in Brazil. Improved internet connectivity has partially mitigated this challenge through telehealth, enabling nursing teams to administer treatment under remote physician supervision, in accordance with Brazilian legislation [16]. However, the arrival of newly hired and often untrained physicians has introduced challenges, including disagreements with more experienced nursing staff. These tensions, driven by differences in experience and familiarity with local protocols, have been managed through team-based discussions and collective decision-making within health units.

The study received prior approval from community leaders, coordinators of the ISHDs, and the CONDISI. In addition, written informed consent was obtained from all participants, either in Portuguese or in Indigenous languages when preferred for better understanding. Participants were informed of their right to withdraw consent at any time without any consequences.

### Privacy and confidentiality

All researchers handling confidential information had clearly established responsibilities to maintain confidentiality, formalized through institutional policies and professional codes of conduct. All data processing complied with the Brazilian General Data Protection Law [17] and applicable ethical regulations [18,19]. Sensitive personal data were collected only as necessary for the study objectives. Written informed consent was obtained from all participants, explicitly addressing data use and confidentiality. All information collected for this proposal is kept in a confidential manner separate from any identifying information to ensure patient confidentiality and avoid potential data security risks. After transcriptions and validation of transcript content, every recording was destroyed to avoid any data leakage. Access to SINAN data was granted through a Data Use Agreement approved by CONEP, and such data were received in anonymized form. Electronic data were kept in encrypted, password-protected systems, and physical records were stored in secure facilities. Access was limited to authorized personnel. The research team and institution acted as data controllers/operators and implemented appropriate technical and administrative safeguards, including incident response procedures. Electronic data repository systems are approved by all regulatory bodies.

### Standard of care

All patients received standard-of-care management for snakebite envenoming, ensuring that no participant was deprived of recommended treatment. Care was guided by a culturally adapted clinical practice guideline for community health centers, validated by experts and informed by frontline providers [14]. This included key components such as diagnosis, severity classification, free-of charge antivenom treatment according to the national guidelines, and follow-up. To ensure safety, health care professionals were trained to recognize and manage adverse reactions to antivenom. Although no such cases occurred, teams were prepared to transfer patients to hospital settings to complete antivenom treatment in the event of allergic reactions that precluded continuation of therapy in community health units. The program also implemented risk-based referral strategies, whereby vulnerable patients, such as pregnant women, older adults, and those with comorbidities, received initial treatment locally and were then transferred to higher-level facilities for monitoring. All patients in the intervention group received the appropriate antivenom according to the type of snakebite envenomation, and antivenom dosage was mostly consistent with clinical severity, with no cases of underdosing reported [4].

### Ancillary care

Medical supplies not directly related to the SAVING Program objectives remained under the responsibility of the Indigenous Health Care Subsystem. Essential medicines and supplies, including analgesics, antibiotics, antihistamines, adrenaline, and basic clinical equipment, were part of the standard requirements for Indigenous health units accredited to deliver antivenom treatment [14]. The study did not introduce additional therapeutic interventions, and no material or clinical benefits were offered as incentives, thereby not constituting undue inducement. Ancillary support within routine care included also transportation assistance and provision of food.

### Community and health-system empowerment

The decentralization of antivenom provision was expected to enable the resolution of snakebite cases within the Indigenous territory, increasing local autonomy in care delivery. In terms of service delivery, it is anticipated to enable timely, point-of-care treatment, reducing delays, medical evacuations, and the risk of severe outcomes. For the health workforce, expanded responsibilities at the primary care level are expected to enhance local capacity, while requiring investment to address workforce instability and training needs. Improvements in health information systems are expected to support better case surveillance and notification. The intervention was also expected to strengthen cold-chain infrastructure. Collectively, these changes are expected to improve system responsiveness, continuity, and efficiency of care, while increasing user confidence in local services and consolidating a shift toward autonomous, in-territory care. However, these outcomes will depend on sustained investment in workforce capacity, infrastructure, and information system integration.

## Dissemination phase

### Dissemination of research findings

Dissemination was conducted throughout the research process, with iterative evaluation based on stakeholder engagement, policy uptake, and accessibility, treating it as a continuous and relational process. Outputs included peer-reviewed publications, technical reports, and locally adapted educational materials for policymakers, the scientific community, and Indigenous populations. Policymakers and healthcare professionals at municipal, state, and federal levels, including Indigenous representatives, were engaged through targeted meetings and actionable reports, supported by partnerships with the Brazilian Ministry of Health and the Pan American Health Organization. For the scientific community, findings are being disseminated through open-access publications and conference presentations, enabling critical appraisal and adaptation to other settings. Within Indigenous territories, dissemination relied on community-based partners and culturally grounded channels; barriers such as limited connectivity and linguistic diversity were addressed through in-person engagement and printed and audiovisual materials adapted to local languages. Messaging was co-developed to ensure relevance and comprehension, countering extractive practices. The funders had no role in decision to publish or preparation of the manuscript.

### Data ownership

The ownership of all data generated within this project is collectively held by the research consortium, in accordance with institutional agreements and applicable national and international regulations. Primary data derived from interviews and co-development sessions with Indigenous participants, healthcare professionals, and policymakers are governed with particular attention to ethical principles, including respect for Indigenous data sovereignty and culturally appropriate data stewardship. Quantitative secondary data obtained from public repositories, such as the SINAN database, remain under the terms of their original custodianship. The study team acts as data stewards, responsible for ensuring secure management, anonymization, and appropriate use of the data throughout the project lifecycle. Data sharing and reuse follow established governance frameworks, with access granted through a Data Access Committee that includes both research and community representatives, ensuring transparency, accountability, and alignment with participants’ consent and regulatory requirements.

### Translation of findings into public health action

The findings of this study directly inform public health action by providing robust evidence to support the decentralization of antivenom delivery within Indigenous primary health care settings. The demonstrated improvements in clinically relevant outcomes support the integration of this model into national snakebite care policies. This approach contributes to strengthening health system equity and responsiveness and offers a transferable model for addressing other time-sensitive conditions in remote and underserved populations.

### Scalability and sustainability

Engagement with policymakers supports integration into health systems aligned with Indigenous priorities. The model’s scalability and sustainability are reinforced by its feasibility and integration within Indigenous health structures, with standardized criteria and culturally adapted training enabling replication in similar remote settings. Sustainability is further supported by leveraging existing systems, such as the vaccine cold chain. The intervention’s expansion has already been approved for 14 additional ISHD of Manaus and Alto Solimões, advancing decentralized antivenom distribution in Amazonas [20]. Scale-up remains constrained by challenges such as staff turnover, infrastructure variability, supply instability, and financing gaps, which must be addressed to ensure long-term impact and effective expansion. Telemedicine offers partial solutions but does not address underlying structural issues.

### Benefit sharing

Benefit sharing in this project is grounded in equity, reciprocity, and respect, addressing a context where Indigenous communities bear disproportionate risks while receiving limited benefits from health interventions. The study underscores that research must generate tangible improvements, including timely access to antivenom, strengthened local capacity, and integration of biomedical and traditional care. Decentralized antivenom delivery contributes directly by reducing delays, improving outcomes, and minimizing patient transfers, while training enhances provider autonomy within Indigenous health systems. Benefits are reinforced through shared tools such as clinical guidelines, training materials, and the SAVING Program, as well as inclusive governance involving community participation.

## Final remarks

Snakebite envenoming remains a high-priority neglected tropical disease that disproportionately affects Indigenous and other underserved populations. Although antivenom is an essential medicine and the cornerstone of treatment, timely access remains limited because of geographic, economic, and structural barriers. Ensuring equitable access is therefore both a public health priority and an ethical imperative grounded in the principles of justice and the right to health [21]. The experience of the SAVING Program demonstrates that decentralizing antivenom treatment within Indigenous territories is both an ethically justified and operationally feasible strategy to reduce these inequities by improving timely treatment and clinical outcomes. At the same time, implementation revealed important ethical tensions between standardized protocols and culturally responsive, context-adapted care, emphasizing the need for flexibility grounded in relational autonomy, meaningful community engagement, and shared decision-making. Sustaining and expanding this model will require continued investment in workforce capacity, infrastructure, telehealth, governance, and reliable antivenom supply.

Beyond improving snakebite care, the SAVING Program illustrates how implementation research can catalyze lasting health system transformation when embedded within ethical principles, Indigenous participation, and policy engagement. Future research should evaluate the long-term sustainability, cost-effectiveness, and health system impact of decentralized antivenom delivery, while exploring adaptation of this model to other remote and underserved populations and to other time-sensitive health conditions requiring decentralized access to essential therapies. The ethical lessons generated by the SAVING Program provide a transferable framework for implementation research and health system innovation aimed at advancing equity for Indigenous and other historically marginalized populations worldwide.
